# Supplementary material for: Plant Triterpenoids Regulate Endophyte Community to Promote Medicinal Plant Schisandra sphenanthera Growth and Metabolites Accumulation
Source: J Fungi (Basel). 2021 Sep 23;7(10):788. doi: 10.3390/jof7100788 (PMC8539763; doi:10.3390/jof7100788)
Supplement: Supplementary file 1 [file jof-07-00788-s001.zip › jof-1397864-supplementary.pdf]

Journal of Fungi

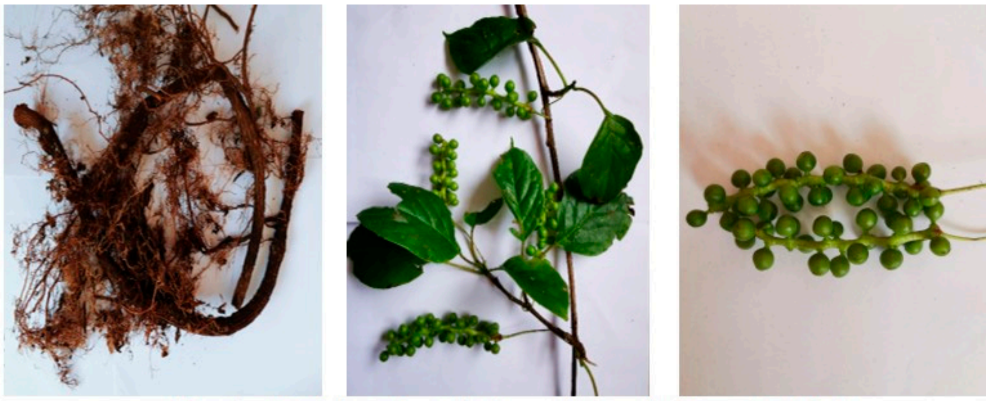

(a) (b) (c)

Figure S1. Morphological pictures of roots (a), stems (b), leaves (b) and fruits (c) of *S. sphenanthera*.

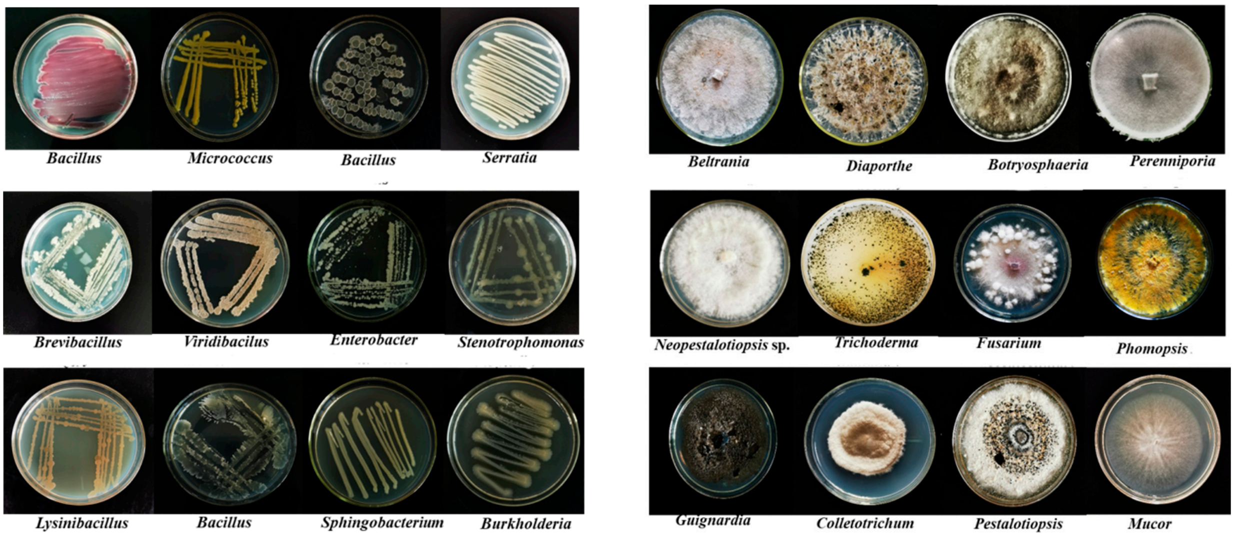

(**a**) (**b**)

Figure S2. Morphological pictures of some representative endophytic bacteria (**a**) and fungi (**b**) from *S. sphenanthera*.

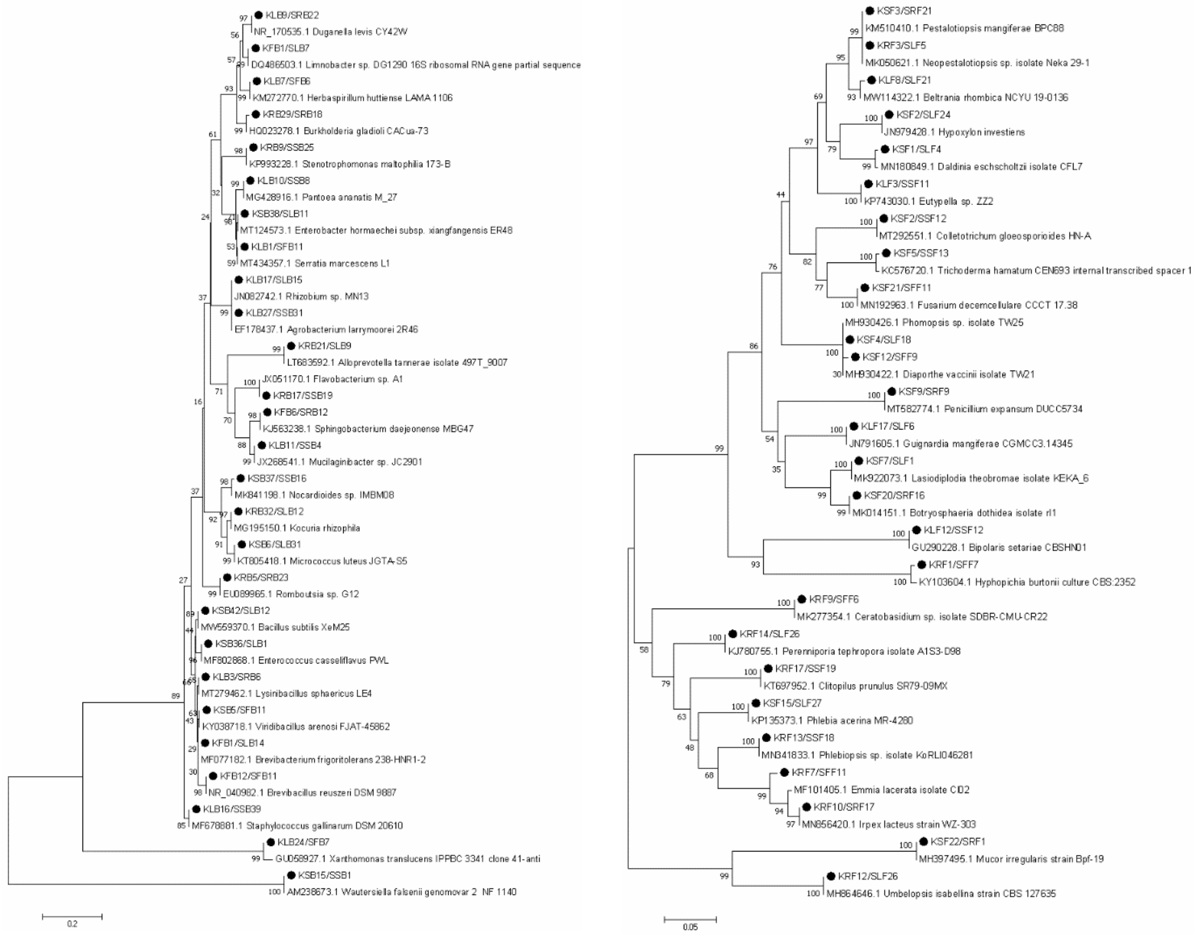

(**a**) (**b**)

Figure S3. Phylogenetic relationships of representative endophytic bacteria and fungi from *S. sphenanthera*.

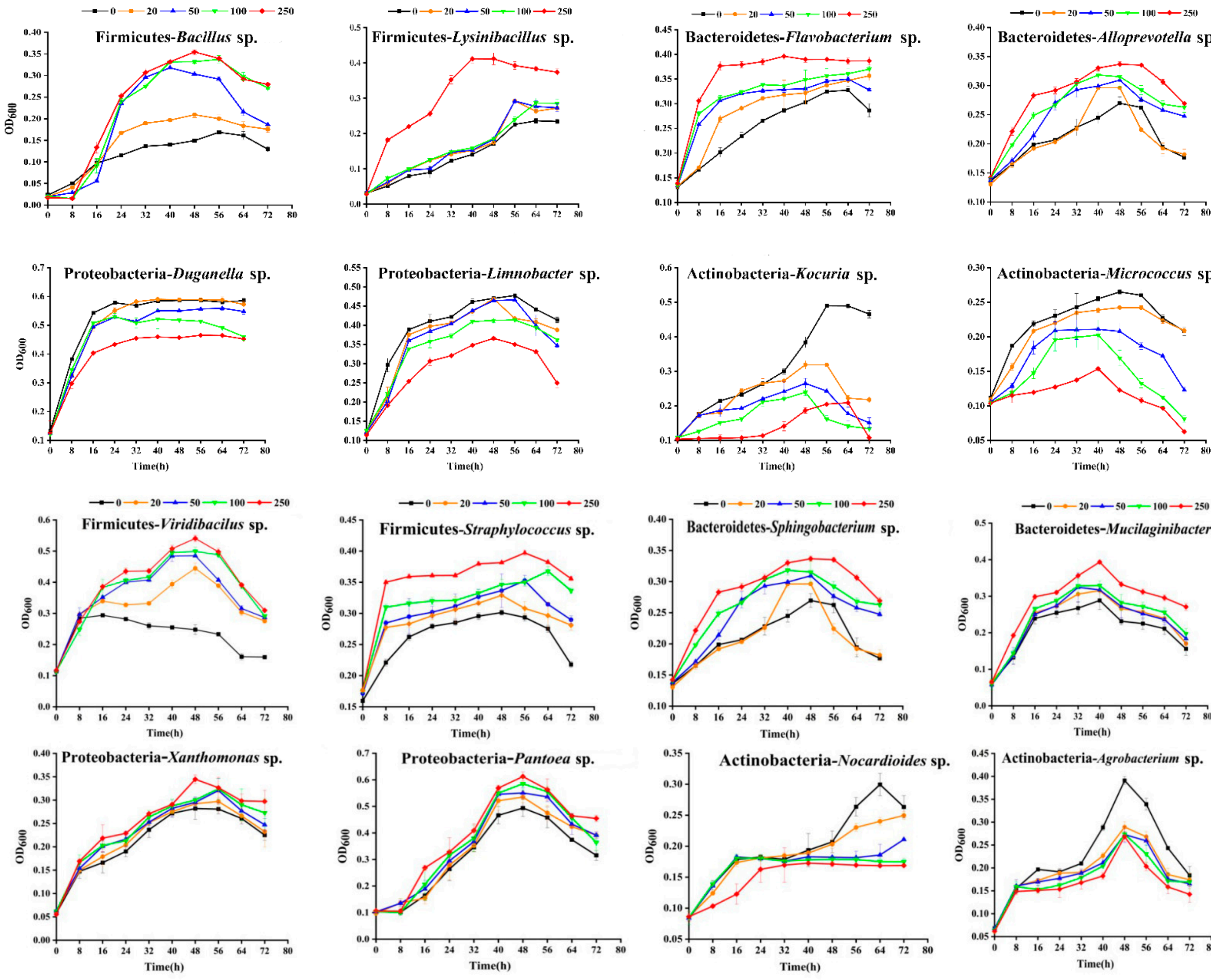

Figure S4. Growth curve of endophytic bacteria under different Mix concentrations. Error bars represent the SEM.

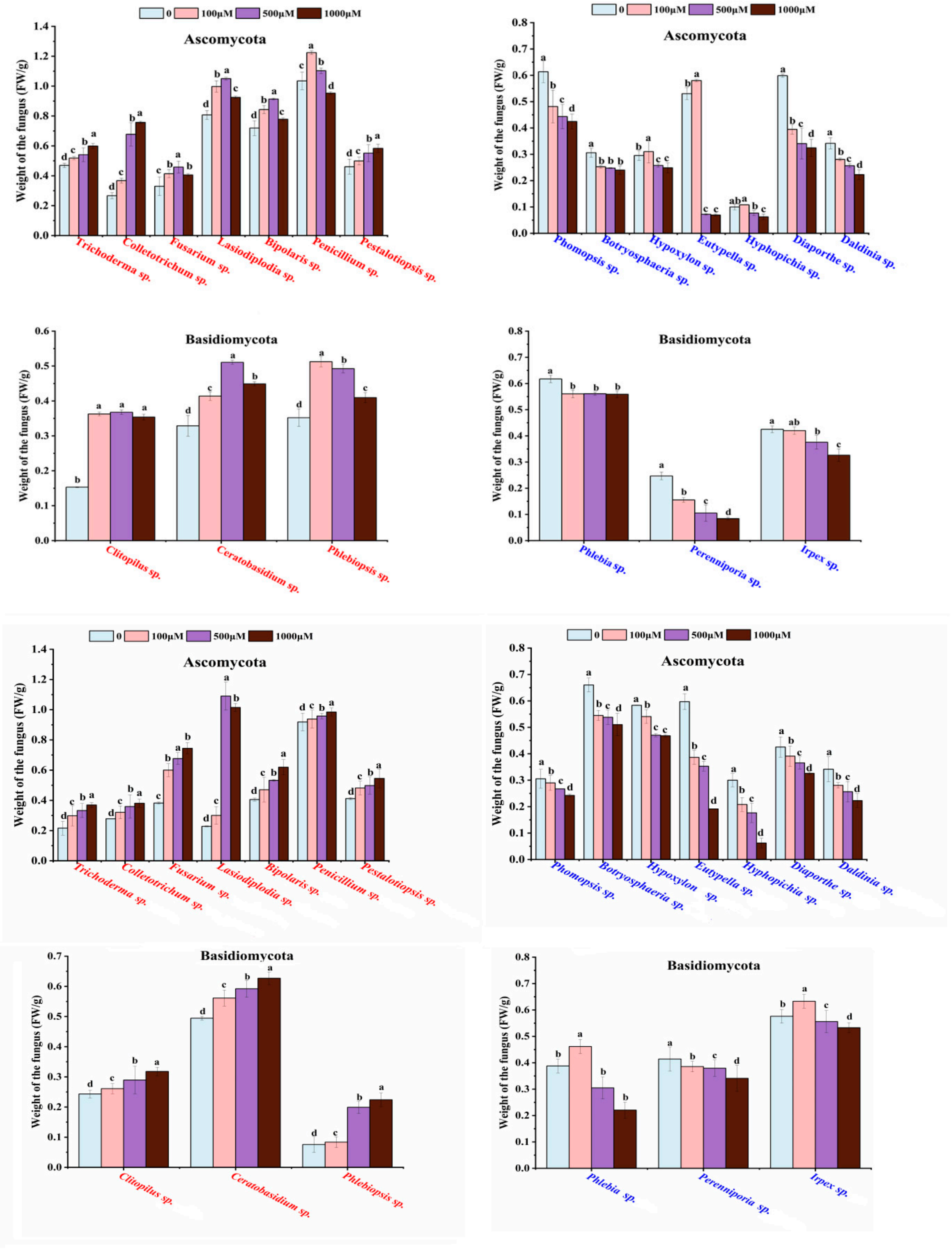

Figure S5. Effects of different concentrations of Mix on the growth of endophytic fungi. Different lowercase letters (a, b, c and d) indicate significant differences between different treatments, one-way ANOVA, Duncan test. Error bars represent the SEM.

(**a**)

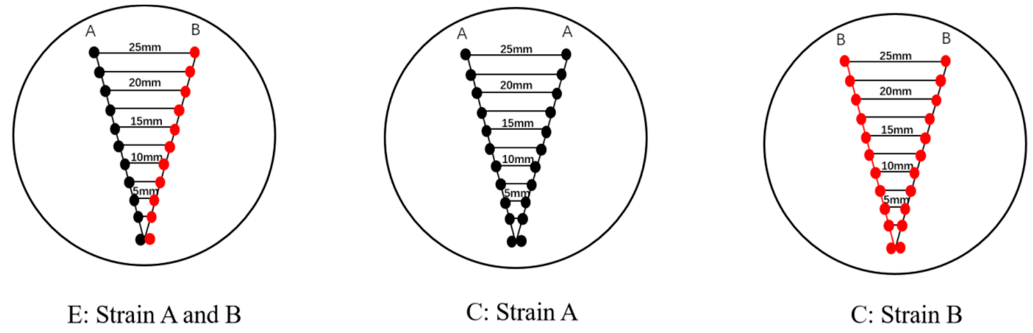

Calculation formula of interaction strength :

$[(D_{25cm\text{-}EA}/D_{10cm\text{-}EA})/\ (D_{25cm\text{-}CA}/D_{10cm\text{-}CA})\text{-}1]$ *100% (The effect of B to A)

$[(D_{25cm\text{-}EB}/D_{10cm\text{-}EB})/\ (D_{25cm\text{-}CB}/D_{10cm\text{-}CB})\text{-}1]$ *100% (The effect of A to B)

(**b**)

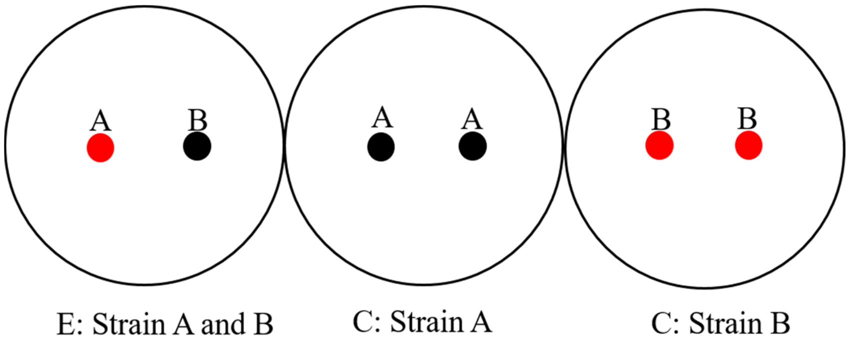

Calculation formula of interaction strength:

$(S_{EA}/S_{CA}\text{-}1)$ *100% (The effect of B to A)

$(S_{EB}/S_{CB}\text{-}1)$ *100% (The effect of A to B)

(**c**)

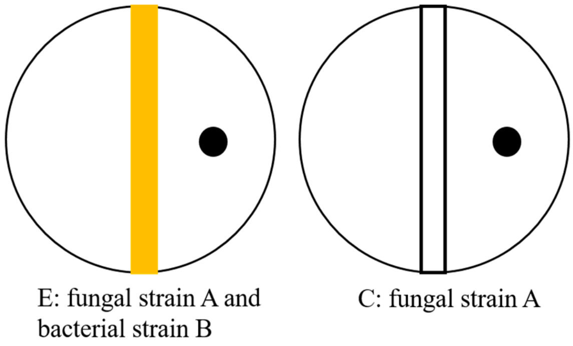

Calculation formula of interaction strength:

$(S_{EA}/\ S_{CA}\text{-}1)$ *100%

Figure S6. Diagram of plate interaction of endophytic bacteria and fungi. (a) Interaction of bacterial and bacterial strains. (b) Interaction of fungal and fungal strains. (c) Interaction of bacterial and fungal strains. Three biological replicates per treatment were used for analysis.

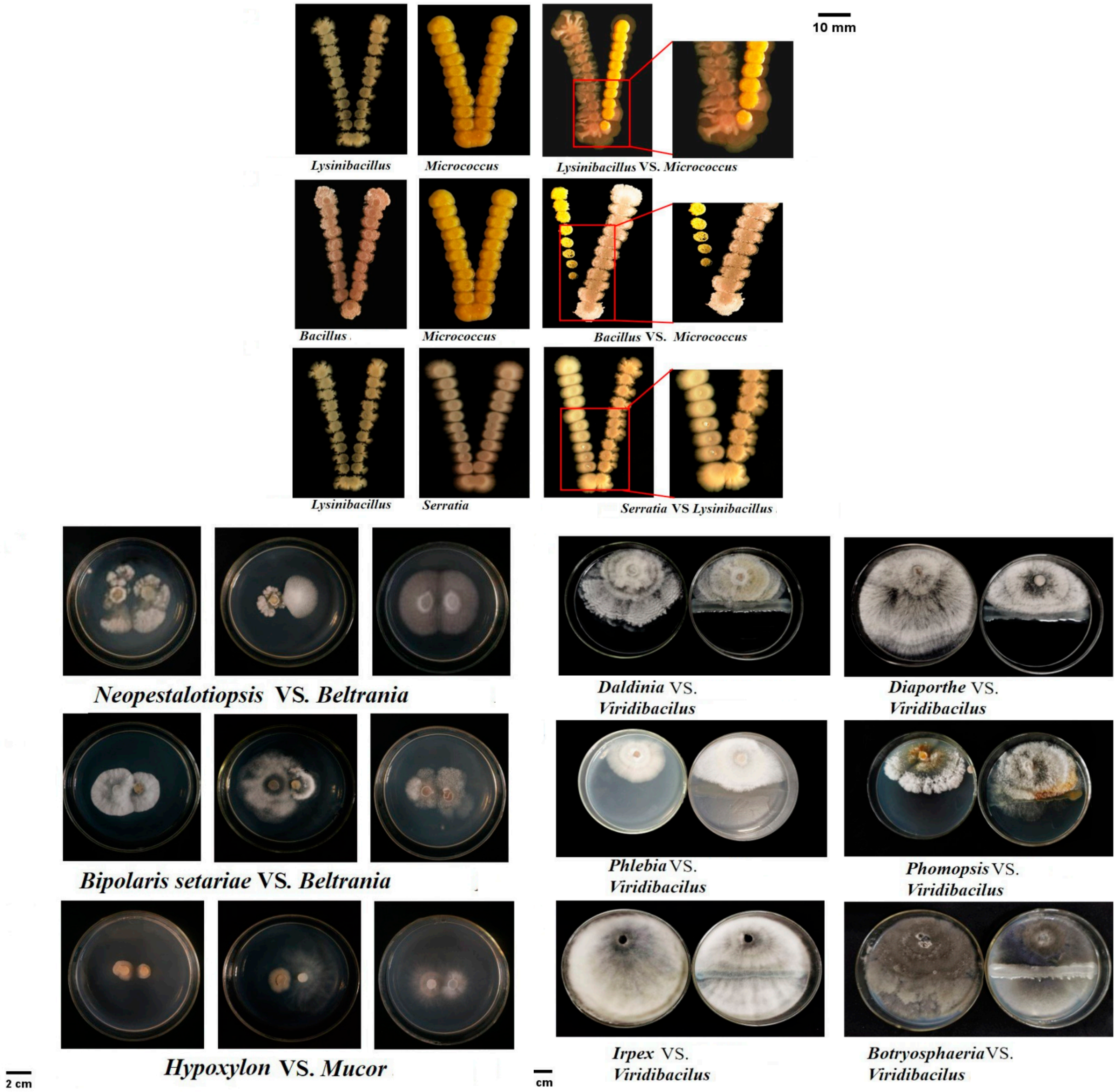

Figure S7. Plate interaction between representative endophytes. (**a**-**c**) Plate interaction between representative endophytic bacteria. A-C represent the promotion, inhibition and no significant effect of the interaction between endophytic bacteria, respectively. (**d**-**f**) Plate interaction between representative endophytic fungi. D-F represent the inhibition, promotion and no significant effect of the interaction between endophytic bacteria, respectively. (**g**-**i**) Plate interaction between representative endophytic bacteria and endophytic fungi. Figures G-I represent the inhibition, promotion and no significant effect of the interaction between endophytic bacteria and fungi, respectively.

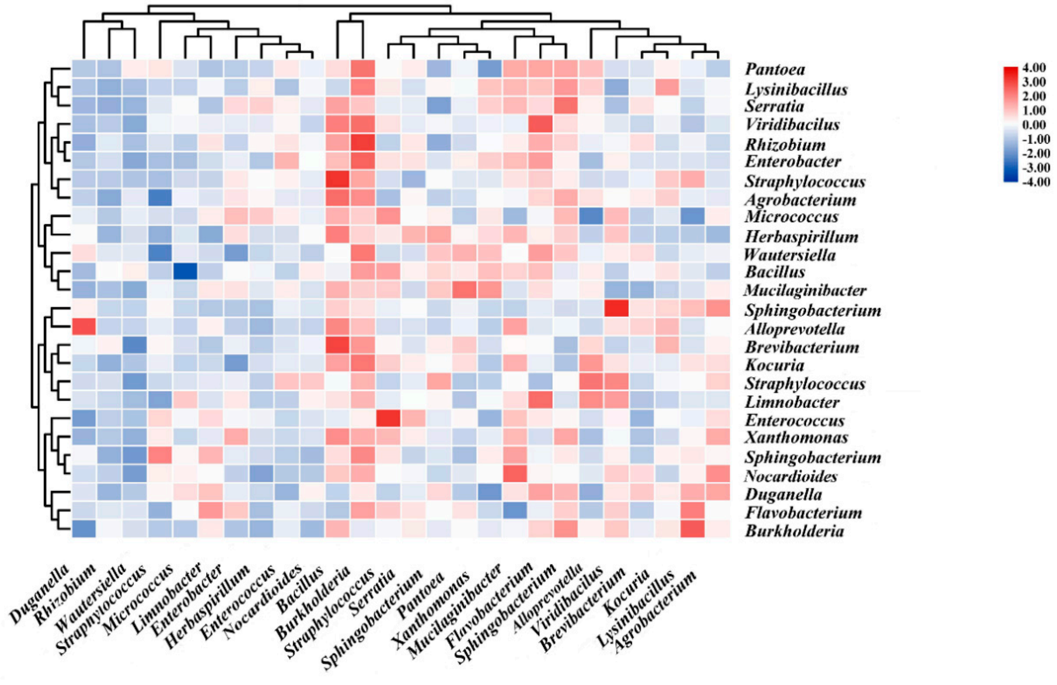

Figure S8. Cluster analysis heat map of interactions at the taxonomic level of the bacterial genus.

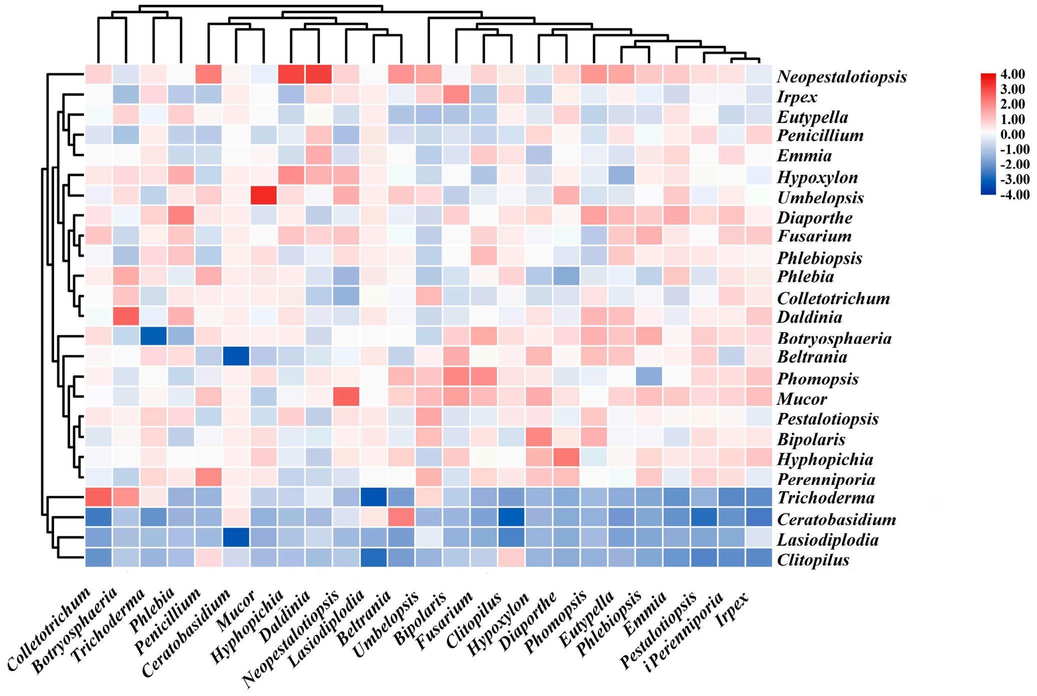

Figure S9. Cluster analysis heat map of interactions at the taxonomic level of the fungal genus.

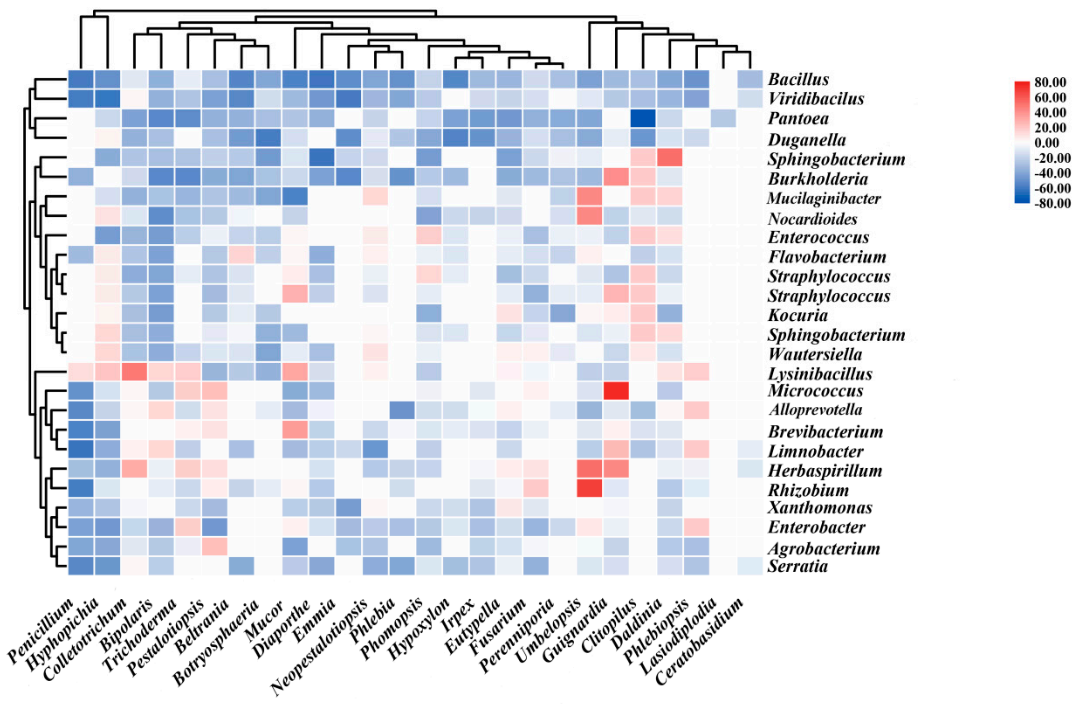

Figure S10. Cluster analysis heat map of interactions at the taxonomic level between the bacterial and fungal genus.

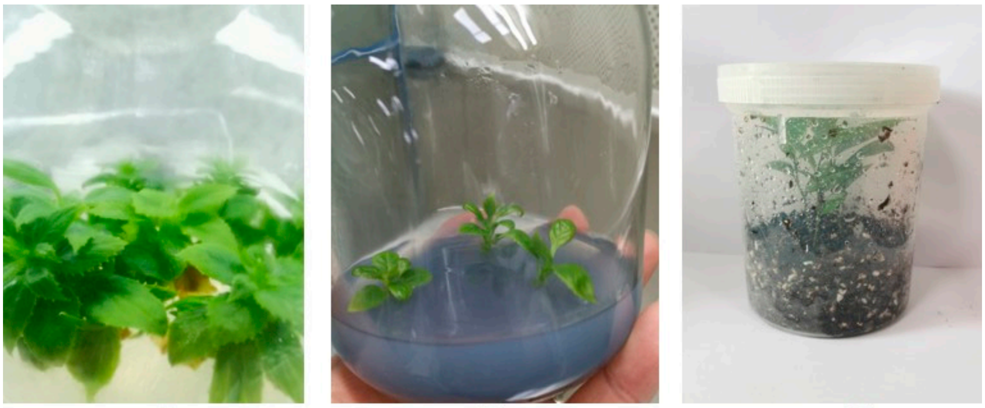

(**a**) (**b**) (**c**)

Figure S11. Co-culture system of synthetic communities and *S. sphenanthera*. (**a**). Tissue culture and proliferation of *S. sphenanthera*. (**b**). Rooting culture of *S. sphenanthera*. (**c**). Co-culture of synthetic communities and tissue culture *S. sphenanthera*.

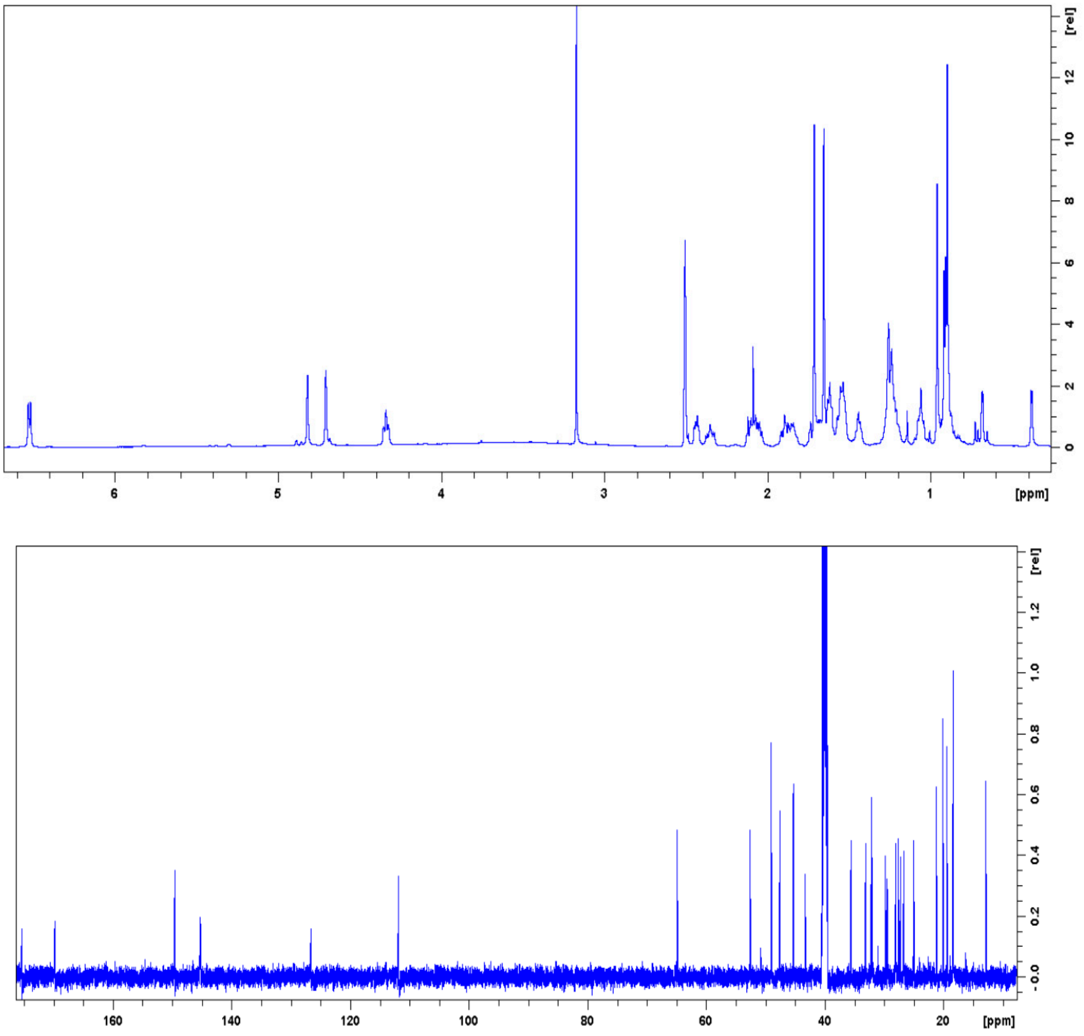

Figure S12. ^{1}H-NMR and ^{13}C-NMR spectrum of nigranoic acid (S-2).

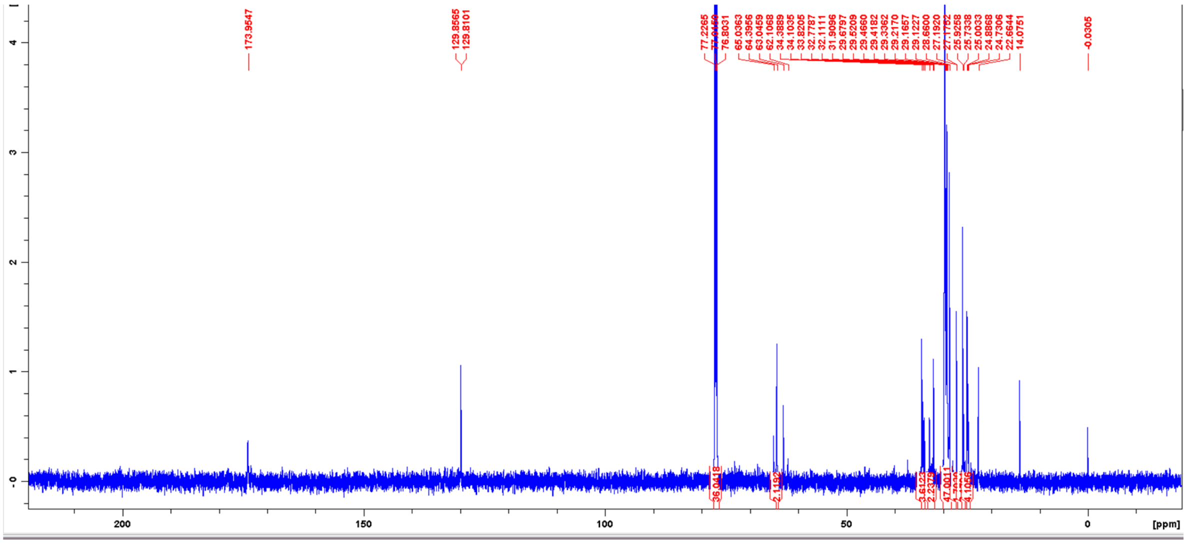

Figure S13. ^{1}H-NMR and ^{13}C-NMR spectrum of iso-anwuweizicacid (S-1).

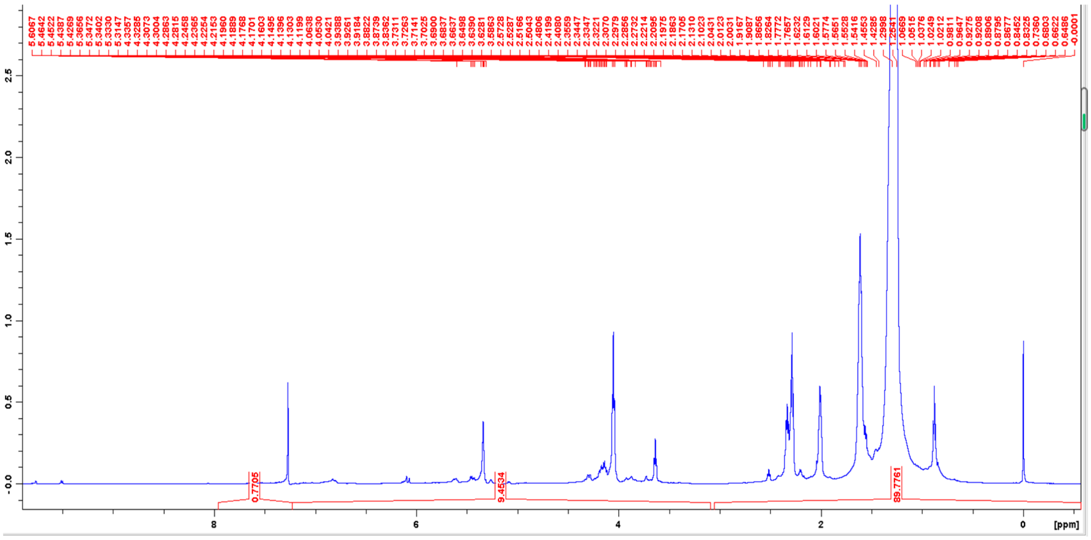

Figure S14. ^{1}H-NMR and ^{13}C-NMR spectrum of micrandilactone C (S-3).

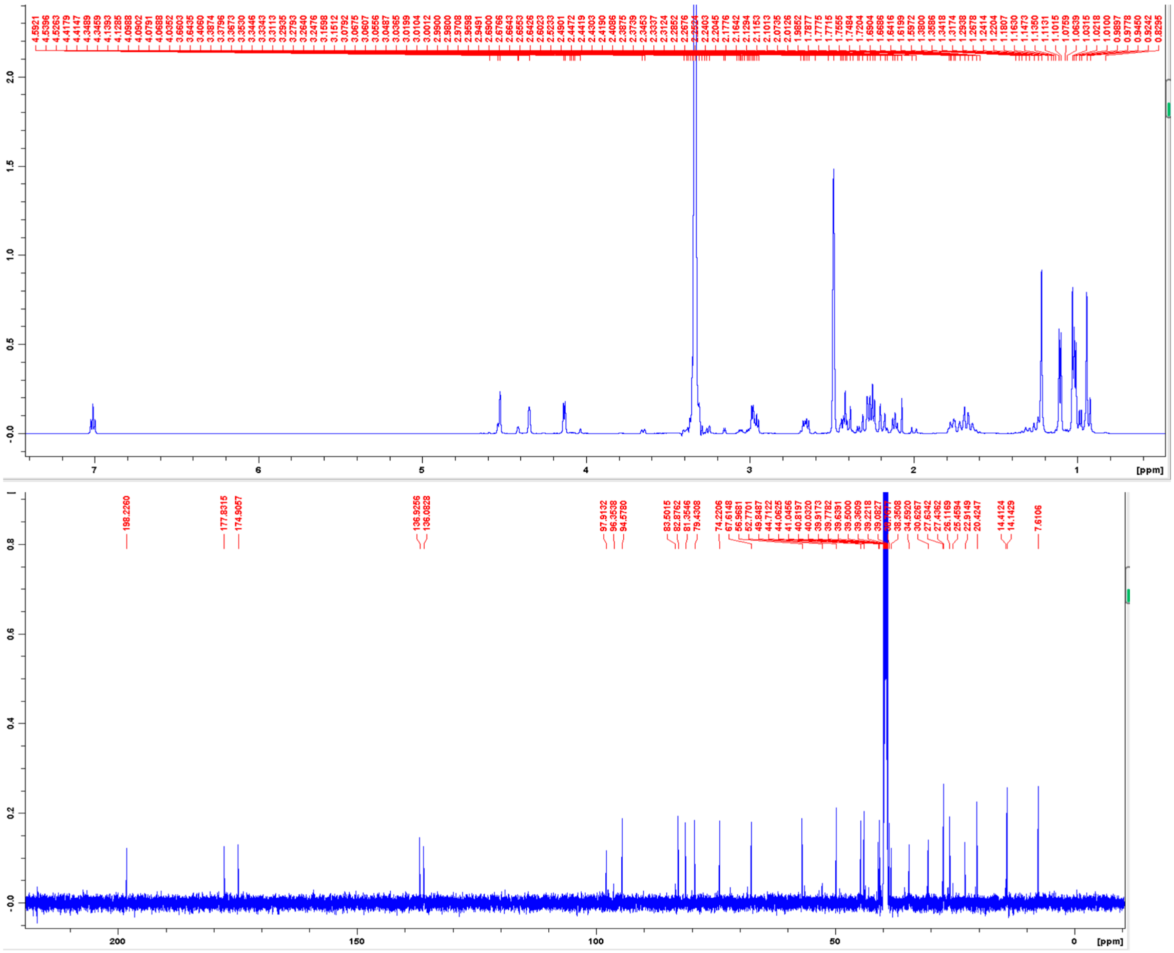

Figure S15. ^{1}H-NMR and ^{13}C-NMR spectrum of lancifodilactone D (S-4).

Table S1. Isolation culture media of endophytic bacteria and fungi.

Type		Composition
bacteria	**TSB**	TSB: Casein 17g, Soybean peptone 3g, NaCl 5g, K_2HPO_4 2.5g, Glucose 2.5g, Agar 20g, pH 7.0
	**TYG**	TYG: Tryptone 1g, Yeast extract 1g, Glucose 0.5g, KCl 6.34 g, NaCl 1.2 g, MgSO4□7H2O 0.25 g, K_2HPO_4 0.13 g, $CaCl_2$□$2H_2O$ 0.22 g, K_2SO_4 0.17 g, Na_2SO_4 2.4 g, $NaHCO_3$ 0.5 g, Na_2CO_3 0.09 g, Fe□ EDTA 0.07 g, Agar 20 g, pH: 7.0
	**YEM**	YEM: Yeast extract 0.5g, Mannitol 5g, K_2HPO_4 0.5 g, $MgSO_4$□ 7H2O 0.2 g, NaCl 0.1 g, Agar 20 g, pH: 7.0
	**TWYE**	TWYE: Yeast extract 0.25g, K_2HPO_4 0.5 g, Agar 18 g, pH: 7.0
	**MYX**	MYX: Sodium glutamate 5 g, Yeast extract 1 g, $MgSO_4$□$7H_2O$ 1 g, Glucose 2 g, Agar 20 g, pH: 7.0
	**MM+MeOH**	MM+MeOH: NH_4Cl 1.62 g, $MgSO_4$□$7H_2O$ 0.2 g, K_2HPO_4 2.4 g, NaH_2PO_4□ $2H_2O$ 1.1 g, Methanol 5 ml, Na_2EDTA□$2H_2O$ 15 mg, $FeSO_4$□$7H_2O$ 3.0 mg, $ZnSO_4$□$7H_2O$ 4.5 mg, $CoCl_2$□$6H_2O$ 3.0 mg, $MnCl_2$ 0.64 mg, H_3BO_3 1.0 mg, Na_2MoO_4□$2H_2O$ 0.4 mg, $CuSO_4$□$5H_2O$ 0.3 mg, $CaCl_2$□$2H_2O$ 3.0 mg, Agar 15 g, pH: 7.1
	**R2A**	R2A: Acid hydrolyzed casein 0.5 g, Yeast extract 0.5 g, Peptone 0.5g, Starch 0.5 g, K_2HPO_4 0.3 g, $MgSO_4$ 0.024 g, Sodium pyruvate 0.3 g, Agar 15 g, pH: 7.2
	**MMF**	MMF: KH_2PO_4 0.1 g, $(NH_4)2SO_4$ 0.1 g, $MgSO_4.7H_2O$ 0.1 g, Metal salt solution 1 mL/L, Glucose 0.05%, pH 5.0
	**GAM**	GAM: Urea peptone 15 g, Soybean peptone 3.0 g, Digested serum powder 13.5 g, Yeast powder 5.0 g, Beef powder 2.0g, Bovine liver powder 1.2 g, Glucose 3.0 g, KH_2PO_4 2.5 g, NaCl 3.0 g, Soluble starch 3.0 g, L-cysteine 0.3 g, Peptone 10.0 g, Sodium thioacetate 0.15 g, Agar 15.0 g, autoclave at 115℃ for 15 min, cool to 50℃, add kanamycin 100 mg/L (Final concentration), neomycin sulfate 100 mg/L, vancomycin hydrochloride 1 mg/L, defibrillated rabbit blood 70 ml/L, hemin 2.5 ml/L and 0.1% vitamin K 1 ml/L
fungi	**PDA**	PDA: Potato 200 g, glucose 20 g, agar 15-20 g, distilled water 1 L, natural pH
	**M + PDA**	M + PDA: Glucose 1g; Peptone 0.5 g, $KH_2PO_4{\cdot}3H_2O$ 0.1g, $MgSO_4{\cdot}7H_2O$ 0.05 g, 0.1% Bengal red solution 0.33 ml, Agar 18 g, 100ml distilled water, natural pH, add 2ml 2% sodium deoxycholate solution and 0.33ml Streptomycin Solution (100 ug/mL) after sterilization
	**CMM**	CMM: $NaNO_3$ 3 g, $KH2PO_4{\cdot}3H_2O$ 1 g, $MgSO_4{\cdot}7H_2O$ 0.5 g, KCl 0.5 g, $FeSO_4$ 0.01g, Sucrose 30 g, Agar 20 g, Distilled water 1L

Table S2. Plant growth promoting ability of representative endophytic bacteria.

Type	Species	DPPH	IAA	P-solubilization	Siderophore
		(%)	(mg/L)	(mg/L)	(%)
TPB	***Bacillus subtilis***	65.72 ±8.19	4.35±0.15	40.76±0.06	44.68±2.88
	Lysinibacillus sphaericus	22.58±0.85	4.53±0.04	129.78±0.57	0.00±0.00
	Viridibacilus arenosi	76.76±3.76	17.55±0.05	42.72±0.64	29.45±1.24
	Straphylococcus sciuri	24.18±2.07	20.00±1.23	110.33±1.60	10.89±2.83
	***Flavobacterium* sp.**	30.49±4.71	35.86±0.20	52.29±2.67	0.00±0.00
	***Mucilaginibacter* sp.**	26.47±3.40	0.73±0.25	76.27±3.31	41.22±0.19
	***Alloprevotella* sp.**	13.45±4.24	32.06±0.90	40.83±0.38	10.80±0.75
	Sphingobacterium daejeonense	28.28±0.83	66.30±0.12	81.83±0.56	7.58±3.60
	Burkholderia gladioli	17.71±0.28	59.61±0.80	72.06±4.23	4.78±0.43
	Xanthomonas arboricola	81.84±10.72	23.80±1.41	52.54±1.46	25.18±2.03
	Pantoea ananatis	78.22±1.97	9.47±0.26	75.73±3.87	41.28±0.64
	Serratia marcescens	22.28±3.47	59.66±1.03	137.87±5.04	10.83±0.03
TNB	***Straphylococcus gallinarum***	19.63±0.84	0.00±0.00	98.06±3.86	0.00±0.00
	Enterococcus casseliflavus	89.76±0.98	0.00±0.00	87.69±0.57	8.90±2.58
	Brevibacillus reuszeri	77.22±2.02	10.89±0.26	58.32±0.80	0.00±0.00

Wautersiella falsenii	36.60±14.51	40.80±0.72	50.44±1.05	6.50±1.70
***Alloprevotella* sp.**	22.84±1.05	3.13±0.05	49.13±0.38	43.84±2.32
Brevibacterium frigoritolerans	50.72±5.52	6.06±0.14	22.65±0.69	0.00±0.00
Kocuria rhizophila	29.92±2.67	66.30±0.26	85.75±3.32	14.38±1.49
Micrococcus luteus	80.17±34.59	59.61±1.07	32.96±0.76	2.56±0.39
***Rhizobium* sp.**	47.17±8.05	16.99±0.12	23.47±0.55	0.00±0.00
Agrobacterium larrymoorei	49.90±7.12	14.23±0.29	35.96±0.56	0.00±0.00
Herbaspirillum huttiense	32.79±5.67	12.38±1.27	22.86±0.42	0.00±0.00
Duganella ruvus	18.49±2.21	28.00±0.08	49.42±3.27	14.72±7.47
***Limnobacter* sp.**	65.88±1.11	21.15±0.12	103.925.04	0.00±0.00
Stenotrophomonas maltophilia	57.76±21.18	3.70±0.20	82.69±0.29	0.00±0.00
Enterobacter hormaechei	66.15±3.10	50.20±1.70	33.47±2.78	46.54±1.45

Data are means of three replicates ± SD.

Table S3. Plant growth promoting ability of 26 representative endophytic fungi.

Type	Species	DPPH	IAA	P-solubilization	Siderophore
		(%)	(mg/L)	(mg/L)	(%)
TPF	***Pestalotiopsis microspora***	71.91±1.12	0.00±0.00	81.20±0.84	51.02±2.29
	Trichoderma hamatum	92.29±0.80	3.62±0.25	44.63±1.78	43.47±0.61
	Colletotrichum gloeosporioides	64.87±3.07	11.90±0.19	24.10±0.21	0.00±0.00
	Fusarium decemcellulare	78.63±4.72	3.52±0.34	14.49±2.09	35.26±2.47
	Lasiodiplodia theobromae	77.75±3.30	19.15±3.68	54.85±2.37	34.02±0.88
	Bipolaris setariae	66.89±4.90	8.15±0.22	18.64±0.17	21.78±1.78
	Penicillium crutosum	35.05±7.62	8.32±0.83	175.62±0.77	53.58±0.58
	Clitopilus prunulus	96.03±1.75	0.00±0.00	10.23±0.08	0.00±0.00
	Ceratobasidium sp.	96.03±1.46	0.00±0.00	71.76±1.96	5.82±1.76
	***Phlebiopsis* sp.**	98.74±29.83	2.11±0.13	159.54±1.29	0.00±0.00
	Umbelopsis isabellina	84.87±19.03	15.36±0.11	99.54±0.13	0.00±0.00
TNF	***Neopestalotiopsis* sp.**	55.32±8.30	10.163±0.38	14.55±1.68	36.36±1.92
	Beltrania rhombica	96.69±0.45	6.39±0.26	62.16±0.31	23.98±0.22
	Guignardia mangiferae	26.71±3.37	0.36±0.08	29.87±0.40	36.99±5.85
	***Phomopsis* sp.**	65.68±5.45	2.53±0.26	19.40±0.09	0.00±0.00
	Botryosphaeria dothidea	82.94±2.27	8.03±0.23	33.70±0.04	43.01±0.25
	Hypoxylon investiens	87.92±7.59	14.13±6.94	51.67±0.27	0.00±0.00
	Eutypella scoparia	76.24±4.01	0.00±0.00	20.07±9.81	0.00±0.00
	Hyphopichia burtonii	22.06±1.54	1.05±0.11	75.23±1.30	0.00±0.00
	Diaporthe vaccinii	71.87±6.32	1.66±0.29	33.48±0.98	45.69±1.22
	Daldinia eschscholtzii	96.03±0.25	2.75±0.13	31.40±0.96	2.67±0.97
	Emmia lacerata	36.29±2.79	0.00±0.00	27.59±1.09	0.00±0.00
	Phlebia acerina	57.21±3.59	6.06±0.43	1.55±0.11	48.58±0.41
	Perenniporia tephropora	32.18±3.57	15.36±1.38	175.62±2.72	30.38±3.77
	Irpex lacteus	35.52±12.37	7.13±0.16	157.64±3.68	9.77±1.08
	Mucor irregularis	92.28±0.79	19.08±1.93	24.03±0.03	0.00±0.00

Data are means of three replicates ± SD.

Table S4. manufacturers of chemicals and solvents.

Chemicals and solvents	manufacturers
EtOH	Kelong chemical reagent factory in Chengdu
Acetone	Kelong chemical reagent factory in Chengdu
MCI	Mitsubishi Chemical Corporation
MS	Beijing QiWei YiCheng Tech.,Ltd.(Qwbio)

BA	Beijing QiWei YiCheng Tech.,Ltd.(Qwbio)
IBA	Beijing QiWei YiCheng Tech.,Ltd.(Qwbio)
DPPH	Solarbio (Beijing)
NaClO	sigma-aldrich (Shanghai)
95% alcohol	Chongqing tixin Chemical Co., Ltd
PDA	Chongqing tixin Chemical Co., Ltd
M + PDA	Chongqing tixin Chemical Co., Ltd
CMM	Chongqing tixin Chemical Co., Ltd
TSB	Chongqing tixin Chemical Co., Ltd
TYG	Chongqing tixin Chemical Co., Ltd
YEM	Chongqing tixin Chemical Co., Ltd
TWYE	Chongqing tixin Chemical Co., Ltd
MYX	Chongqing tixin Chemical Co., Ltd
MM+MeOH	Chongqing tixin Chemical Co., Ltd
R2A	Chongqing tixin Chemical Co., Ltd
MMF	Chongqing tixin Chemical Co., Ltd
GAM	Chongqing tixin Chemical Co., Ltd

Table S5. list of abbreviations.

Abbreviations	Full name
DPPH	1, 1-diphenyl-2-picrylhydrazyl radical 1
IAA	indole 3-acetic acid
CTAB	Cetyl trimethyl ammonium bromide
ITS	Internal transcribed spacer
TSB	Tryptone, Soya Broth
TYG	Tryptone Yeast extract Glucose
YEM	Yeast Extract Manitol
TWYE	Tap Water Yeast Extract
MM+MeOH	Minimal media containing Methanol
PDA	Potato Dextrose Agar
PDB	Potato dextrose broth
M + PDA	Martin Broth,Modified
TLC	Thin layer chromatography
MS	Murashig and Skoog Medium
BA	Benzylaminopurine
IBA	Indolebutyric acid
SynCom	Synthetic community
SEM	Scanning electron microscope
PGPT	Plant growth promotion traits
